# Pseudoprogression and peritumoral edema due to intratumoral necrosis after Gamma knife radiosurgery for meningioma

**DOI:** 10.1038/s41598-022-17813-9

**Published:** 2022-08-11

**Authors:** In-Ho Jung, Kyung Won Chang, So Hee Park, Hyun Ho Jung, Jong Hee Chang, Jin Woo Chang, Won Seok Chang

**Affiliations:** 1 Department of Neurosurgery, Brain Research Institute, Yonsei University College of Medicine, Seoul, 03722 Republic of Korea; 2 Department of Neurosurgery, Brain Tumor Center, Yonsei University College of Medicine, Seoul, 03722 Republic of Korea; 3 Department of Neurosurgery, Dankook University College of Medicine, Cheonan, Republic of Korea

**Keywords:** Cancer, Neuroscience, Medical research, Neurology, Oncology

## Abstract

Peritumoral cerebral edema is reported to be a side effect that can occur after stereotactic radiosurgery. We aimed to determine whether intratumoral necrosis (ITN) is a risk factor for peritumoral edema (PTE) when gamma knife radiosurgery (GKRS) is performed in patients with meningioma. In addition, we propose the concept of pseudoprogression: a temporary volume expansion that can occur after GKRS in the natural course of meningioma with ITN. This retrospective study included 127 patients who underwent GKRS for convexity meningioma between January 2019 and December 2020. Risk factors for PTE and ITN were investigated using logistic regression analysis. Analysis of variance was used to determine whether changes in tumor volume were statistically significant. After GKRS, ITN was observed in 34 (26.8%) patients, and PTE was observed in 10 (7.9%) patients. When postoperative ITN occurred after GKRS, the incidence of postoperative PTE was 18.970-fold (p = 0.009) greater. When a 70% dose volume ≥ 1 cc was used, the possibility of ITN was 5.892-fold (p < 0.001) higher. On average, meningiomas with ITN increased in volume by 128.5% at 6 months after GKRS and then decreased to 94.6% at 12 months. When performing GKRS in meningioma, a 70% dose volume ≥ 1 cc is a risk factor for ITN. At 6 months after GKRS, meningiomas with ITN may experience a transient volume expansion and PTE, which are characteristics of pseudoprogression. These characteristics typically improve at 12 months following GKRS.

## Introduction

Stereotactic radiosurgery is an established treatment and is as applicable to patients with meningioma as surgical resection is. It has the advantages of not requiring general anesthesia, being less invasive, and requiring a shorter hospital stay. Moreover, radiosurgery shows a sufficient local tumor control rate and an acceptable complication rate comparable to resection^1–5^.

However, peritumoral cerebral edema has been reported as a side effect of stereotactic radiosurgery^6–10^. Traditionally, peritumoral edema (PTE) is considered to be caused by radiation-induced injury to the brain parenchyma. However, it is difficult to believe that brain parenchymal injury is caused by the low maximal dose applied to benign tumors, considering that a maximal dose of 130 Gy or more is applied during stereotactic radiosurgical thalamotomy to treat movement disorders^11–13^. Therefore, we investigated other mechanisms that might cause PTE to occur in the early postoperative stage.

We have experienced many cases of PTE occurring after gamma knife radiosurgery (GKRS) for meningioma. PTE is often accompanied by intratumoral necrosis (ITN), and meningioma with ITN is often accompanied by a transient volume expansion. In this study, we evaluated our experiences statistically to determine whether ITN is a risk factor for PTE when GKRS is performed in patients with meningioma. In addition, we propose evidence of pseudoprogression: a temporary volume expansion that can occur after GKRS in the natural course of meningioma with ITN.

## Methods

The inclusion criteria are as follows; Patients who underwent GKRS for convexity meningioma between January 2019 and December 2020 and who had follow-up MRI to evaluate the perioperative tumor changes. Meningioma with falx, parasaggital, and skull base locations were excluded from this retrospective study. We evaluated risk factors for ITN and PTE by analyzing the demographics, tumor characteristics, and radiosurgical prescription parameters of the patients. In addition, the tumor volumes before GKRS, and postoperatively after 6 and 12 months, were measured to chart the natural course of the tumor after ITN. This study was conducted in accordance with the Declaration of Helsinki and was approved by Institutional Review Board of Severance hospital (4-2021-1190). The requirement to obtain written consent from the patients was waived because this was a retrospective study.

### Gamma knife radiosurgery

On the day of treatment, each patient was affixed with the Leksell stereotactic frame G (Elekta Instruments AB; Stockholm, Sweden) and administered local anesthesia. After the stereotactic frame was fixed to the head, the patients underwent magnetic resonance imaging (MRI; 1.5 Tesla Philips Achieva). The MRI for GKRS included gadolinium-enhanced T1-weighted image with a slice thickness of 1.5 mm and T2-weighted image with a slice thickness of 2.5 mm. Radiosurgical planning was performed using Leksell Gamma Plan version 11.1.0, and the radiosurgery was performed using the Leksell Gamma Knife Perfexion Unit (Elekta AB, Elekta Company; Stockholm, Sweden). The prescription dose was 14 Gy with a median 50% isodose line. However, for large tumors that exceeded 10 cc, the prescription dose could be lowered to 13 Gy at the discretion of the neurosurgeon.

As radiosurgical prescription parameters, the 50% marginal dose, coverage, selectivity, gradient index, and 70% dose volume were investigated. Coverage was defined as the fraction of the tumor volume within the prescribed isodose volume. Selectivity was defined as the fraction of the prescribed isodose volume within the tumor volume. The gradient index was defined as the volume ratio of half the prescribed isodose to the total prescription isodose. The 70% dose volume was defined as the tumor volume that received 70% of the maximal dose.

### Neuroimaging work-up

All patients underwent MRI with gadolinium enhancement before the GKRS, and we determined the tumor characteristics based on this MRI. After GKRS, the patients were scheduled for clinical evaluation and postoperative MRI at 6 and 12 months. In addition, we performed an MRI exam whenever a patient showed neurological deterioration after GKRS. The tumor characteristics included the tumor volume, PTE, ITN, brain–tumor contact-surface index, and tumor/cerebellar peduncle T2-weighted imaging intensity (TCTI) ratio. As described by Hwang et al.^14^, the brain–tumor contact-surface index was defined as the ratio of the tumor base length (dural side) and tumor dome length (parenchymal side) on an MRI slide that showed the maximum cross-sectional area of the tumor. To evaluate tumor consistency, TCTI ratios were calculated according to Smith et al. using T2-weighted MRIs^15–18^. The TCTI ratio was defined as the average intensity value in the tumor region of interest divided by the average intensity value in the ROI drawn within the cerebellar peduncle.

ITN was defined as newly developed necrosis within a tumor, or aggravation of pre-existing necrosis, on T1-weighted images obtained after GKRS. PTE was defined as a newly developed high-signal change around a tumor, or aggravation of pre-existing PTE, on T2-weighted images obtained after GKRS.

### Statistical analysis

All statistical analyses were performed using IBM SPSS Statistics (Version 25.0; IBM; Armonk, NY, USA). The risk factors for PTE and ITN were analyzed using logistic regression analysis. The multivariable regression analysis was carried out using variables having a *p*-value of less than 0.10 in the univariate analysis. Analysis of variance (ANOVA) was performed to determine whether changes in tumor volume were statistically significant. *P*-values < 0.05 were considered statistically significant.

### Ethical approval

This study was conducted in accordance with the Declaration of Helsinki and was approved by Institutional Review Board of Severance hospital, waving the patient's written consent as a retrospective study (4-2021-1190). The requirement for obtaining patient's written consent was waived as this was a retrospective study.

## Results

### Demographics and tumor characteristics

Between January 2019 and December 2020, 127 patients underwent GKRS for convexity meningioma in our institution. Their mean age was 59.6 years and the average volume of the tumors was 1.7 cc. The average follow-up period was 10.3 months. The details of the demographics and the tumor characteristics of the patients are presented in Table 1.

**Table 1:** Demographics and tumor characteristics.

Characteristics	N = 127
**Sex**	
Female	108 (85.0%)
Male	19 (15.0%)
Age (years)	59.6 ± 10.3
Tumor volume, preoperative (cc)	1.7 ± 1.8
TCTI	1.4 ± 0.4
Brain-tumor contact-surface index	0.56 ± 0.13
Peritumoral edema, preoperative	3 (2.4%)

*TCTI* tumor/cerebellar peduncle T2-weighted imaging intensity.

### Radiosurgical prescription parameters

There were 125 (98.4%) patients who had 14 Gy prescribed as the 50% marginal dose. The mean coverage was 98.1 ± 2.7%, the mean selectivity was 70.7 ± 12.5%, and the mean gradient index was 2.76 ± 0.18. The average 70% dose volume was 0.9 ± 0.9 cc, and the average 70% dose ratio was 55.6 ± 8.5%. The radiosurgical prescription parameters are shown in Table 2.

**Table 2:** Radiosurgical prescription parameters.

Characteristics	N = 127
**50% Marginal dose (Gy)**	
14.0	125 (98.4%)
13.5	1 (0.8%)
13.0	1 (0.8%)
Coverage (%)	98.1 ± 2.7
Selectivity (%)	70.7 ± 12.5
Gradient index	2.76 ± 0.18
70% dose volume (cc)	0.9 ± 0.9
70% dose ratio (%)	55.6 ± 8.5

### Radiosurgical outcomes

Following GKRS, ITN was observed in 34 (26.8%) patients, and PTE was observed in 10 (7.9%) patients. Nine of the 10 patients with PTE had ITN. Additionally, 3 of them displayed neurological problems (1 motor weakness, 2 seizures). Mannitol and steroids were provided to all patients who displayed neurological symptoms. All PTEs and symptoms had subsided at six months after GKRS.

The tumor volumes measured in the first follow-up MRI had decreased by 0.6% in the absence of ITN but increased by 24.5% in the presence of ITN. The details of the radiosurgical outcomes are shown in Table 3.

**Table 3:** Radiosurgical outcomes.

Variables	N = 127
Intratumoral necrosis, postoperative	34 (26.8%)
**Tumor volume change (%)**	
Intratumoral necrosis, yes	24.5 ± 22.3
Intratumoral necrosis, no	− 0.6 ± 21.5
Peritumoral edema, postoperative	10 (7.9%)

*TCTI* tumor/cerebellar peduncle T2-weighted imaging intensity.

### Risk factors for postoperative peritumoral edema

To identify risk factors of postoperative PTE, univariable and multivariable logistic regression analyses were performed (Table 4). In the univariable logistic regression analysis, the variables whose *p*-values were < 0.100 included a preoperative tumor volume ≥ 3 cc (Odds ratio [OR] 6.424, 95% confidence interval [CI] 1.570–26.294; *p* = 0.010), preoperative PTE (OR 29.000, 95% CI 2.368–355.096; *p* = 0.008), postoperative ITN (OR 33.120, 95% CI 4.004–273.933; *p* = 0.001), selectivity ≥ 70% (OR 6.485, 95% CI 0.795–52.874; *p* = 0.081), and 70% dose volume ≥ 1 cc (OR 4.350, 95% CI 1.149–16.472; *p* = 0.030). Multivariable logistic regression analysis was performed using these five variables. The only variable showing a significant difference was the presence of postoperative ITN. When postoperative ITN occurred following GKRS, the incidence of postoperative PTE showed an 18.970-fold increase (95% CI: 2.111–170.497; *p* = 0.009). The model results had a Nagelkerke R-squared value of 0.419, which suggests that the model explained 41.9% of the variation in the outcome. The Hosmer–Lemeshow test of the goodness of fit suggested that the model was a good fit to the data, since *p* = 0.131 (*p* > 0.05).

**Table 4:** Univariable and multivariable logistic regression analyses for postoperative peritumoral edema.

Variables	Univariable		Multivariable	
	OR (95% CI)	*p*	OR (95% CI)	*p*
**Sex**				
Female	1.000 (–)	–		
Male	2.705 (0.634–11.553)	0.179		
**Age (years)**	1.043 (0.976–1.115)	0.213		
< 60	1.000 (–)			
≥ 60	2.294 (0.566–9.304)	0.245		
**Tumor volume, preoperative (cc)**				
< 3	1.000 (–)			
≥ 3	6.424 (1.570–26.294)	**0.010**	4.221 (0.799–22.305)	0.090
**TCTI**				
< 1.5	1.000 (–)			
≥ 1.5	2.343 (0.638–8.607)	0.200		
**Brain-tumor contact-surface index**				
< 0.5	1.000 (–)			
≥ 0.5	1.639 (0.331–8.116)	0.545		
**Peritumoral edema, preoperative**	29.000 (2.368–355.096)	**0.008**	11.662 (0.814–167.022)	0.070
**Intratumoral necrosis, postoperative**	33.120 (4.004–273.933)	**0.001**	18.970 (2.111–170.497)	**0.009**
**Coverage**				
< 95%	1.000 (–)	–		
≥ 95%	1.125 (0.132–9.609)	0.914		
**Selectivity**				
< 70%	1.000 (–)	–	1.000 (–)	–
≥ 70%	6.485 (0.795–52.874)	**0.081**	3.979 (0.309–51.249)	0.290
**Gradient index**				
< 2.70	1.000 (–)	–		
≥ 2.70	0.625 (0.171–2.280)	0.477		
**70% dose volume**				
< 1 cc	1.000 (–)	–	1.000 (–)	–
≥ 1 cc	4.350 (1.149–16.472)	**0.030**	0.536 (0.059–4.828)	0.578
**70% dose ratio**				
< 50%	1.000 (–)	–		
≥ 50%	1.639 (0.331–8.116)	0.545		

Significant values are in bold.

*TCTI* tumor/cerebellar peduncle T2-weighted imaging intensity, *OR* odds ratio; *CI* confidence interval.

### Risk factors for intratumoral necrosis

To identify factors that could help predict postoperative ITN, univariable and multivariable logistic regression analyses were performed (Table 5). In the univariable logistic regression analysis, the variables whose *p*-value were < 0.100 were a preoperative tumor volume ≥ 3 cc (OR 5.220, 95% CI 1.695–16.073; *p* = 0.004), selectivity ≥ 70% (OR 2.676, 95% CI 1.097–6.527; *p* = 0.030), gradient index ≥ 2.7 (OR 0.333, 95% CI 0.148–0.749; *p* = 0.008), and 70% dose volume ≥ 1 cc (OR 6.875, 95% CI 2.881–16.406; *p* < 0.001). Multivariable logistic regression analysis was performed using these four variables. The only variable that showed a significant difference was having a 70% dose volume ≥ 1 cc. When the 70% dose volume was ≥ 1 cc, the incidence of ITN showed a 5.892-fold increase (95% CI 2.420–14.349; *p* < 0.001). The model results had a Nagelkerke R-squared value of 0.242, which suggests that the model explained 24.2% of the variation in the outcome. The Hosmer–Lemeshow test of the goodness of fit suggested the model was a good fit to the data, since *p* = 0.947 (*p* > 0.05).

**Table 5:** Univariable and multivariable logistic regression analyses for intratumoral necrosis.

Variables	Univariable		Multivariable	
	OR (95% CI)	*p*	OR (95% CI)	*p*
**Sex**				
Female	1.000 (–)	–		
Male	2.294 (0.834–6.310)	0.108		
**Age** (years)				
< 60	1.000 (–)	–		
≥ 60	1.460 (0.659–3.232)	0.351		
**Tumor volume, preoperative** (cc)				
< 3	1.000 (–)	–		
≥ 3	5.220 (1.695–16.073)	**0.004**	1.118 (0.281–4.452)	0.874
**TCTI**				
< 1.5	1.000 (–)	–		
≥ 1.5	1.055 (0.455–2.449)	0.900		
**Coverage**				
< 95%	1.000 (–)	–		
≥ 95%	1.386 (0.362–5.304)	0.633		
**Selectivity**				
< 70%	1.000 (–)	–	1.000 (–)	–
≥ 70%	2.676 (1.097–6.527)	**0.030**	1.011 (0.969–1.056)	0.603
**Gradient index**				
< 2.70	1.000 (–)	–	1.000 (–)	–
≥ 2.70	0.333 (0.148–0.749)	**0.008**	0.454 (0.188–1.094)	0.078
**70% dose volume**				
< 1 cc	1.000 (––)	–	1.000 (–)	–
≥ 1 cc	6.875 (2.881–16.406)	** < 0.001**	5.892 (2.420–14.349)	** < 0.001**
**70% dose ratio**				
< 50%	1.000 (–)	–		
≥ 50%	0.531 (0.230–1.226)	0.138		

Significant values are in bold.

*TCTI* tumor/cerebellar peduncle T2-weighted imaging intensity, *OR* odds ratio, *CI* confidence interval.

**Figure 1:**
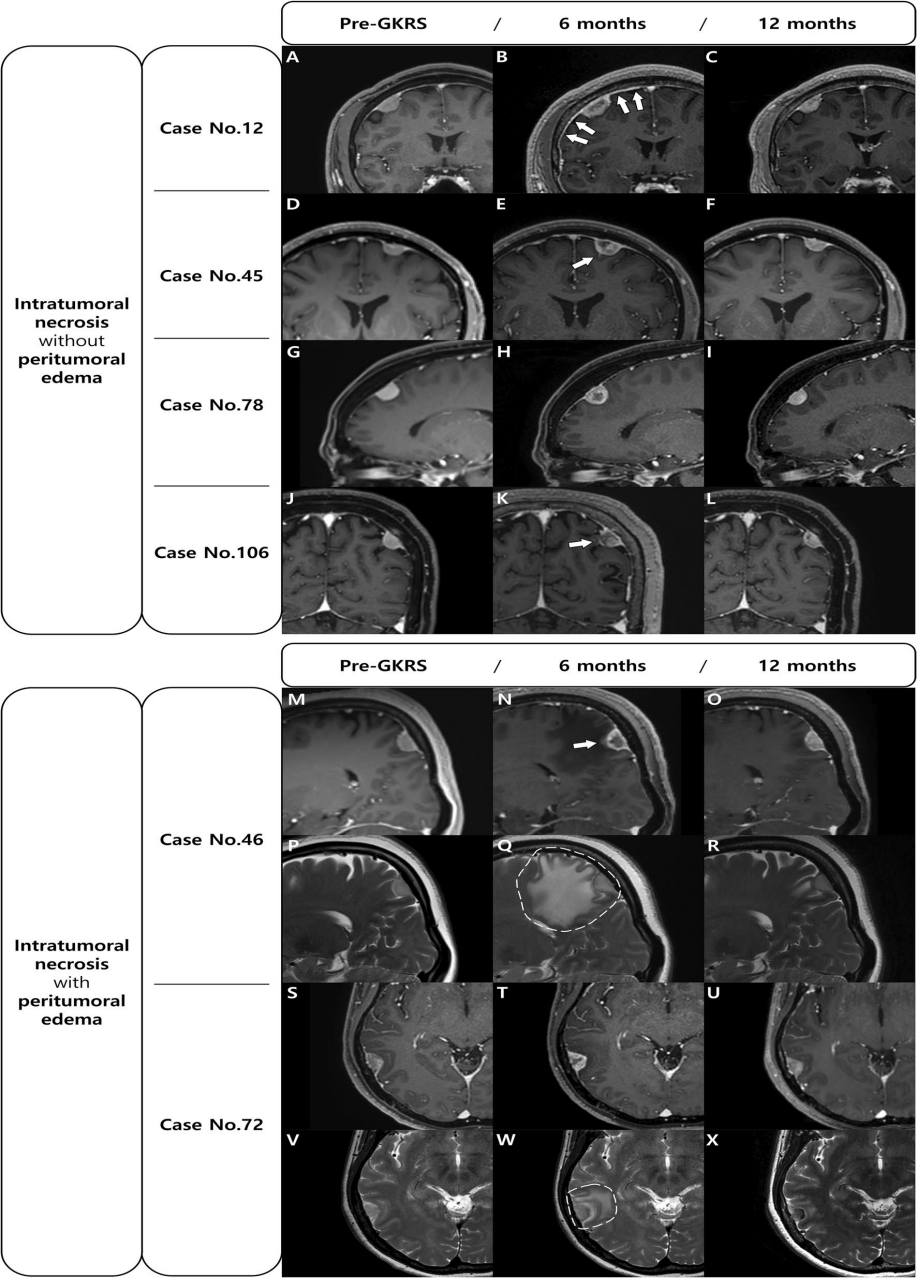
Changes occurring before and after GKRS are shown for 4 cases of ITN without PTE (**A**–**L**) and 2 cases of ITN with PTE (**M**–**X**). All meningiomas with ITN after GKRS show pseudoprogression with a temporary volume expansion on the follow-up MRI at 6 months (**B**,**E**,**H**,**K**,**N**,**Q**,**T**,**W**). ITN may accompany meningeal enhancement (arrows) on T1-weighted images and PTE on T2-weighted images (dotted circles). On the follow-up MRI at 12 months, the ITN has disappeared, the volume is reduced, and the enhancement and PTE are improved. *GKRS* gamma knife radiosurgery; *ITN* intratumoral necrosis, *PTE* peritumoral edema, *MRI* magnetic resonance imaging.

In addition, a dural, arachnoid, or pial enhancement around the tumor was observed in 16 (47.1%) of the 34 patients with meningioma exhibiting ITN (Fig. 1). However, none of the cases of meningioma without ITN showed a meningeal enhancement. There was a significant difference between the two groups (*p* < 0.001); therefore, we suggest that ITN might be associated with meningeal enhancement. Figure 1 shows the changes before and after GKRS for 4 cases of ITN without PTE and 2 cases of ITN with PTE.

### Tumor volume change after gamma knife radiosurgery

**Figure 2:**
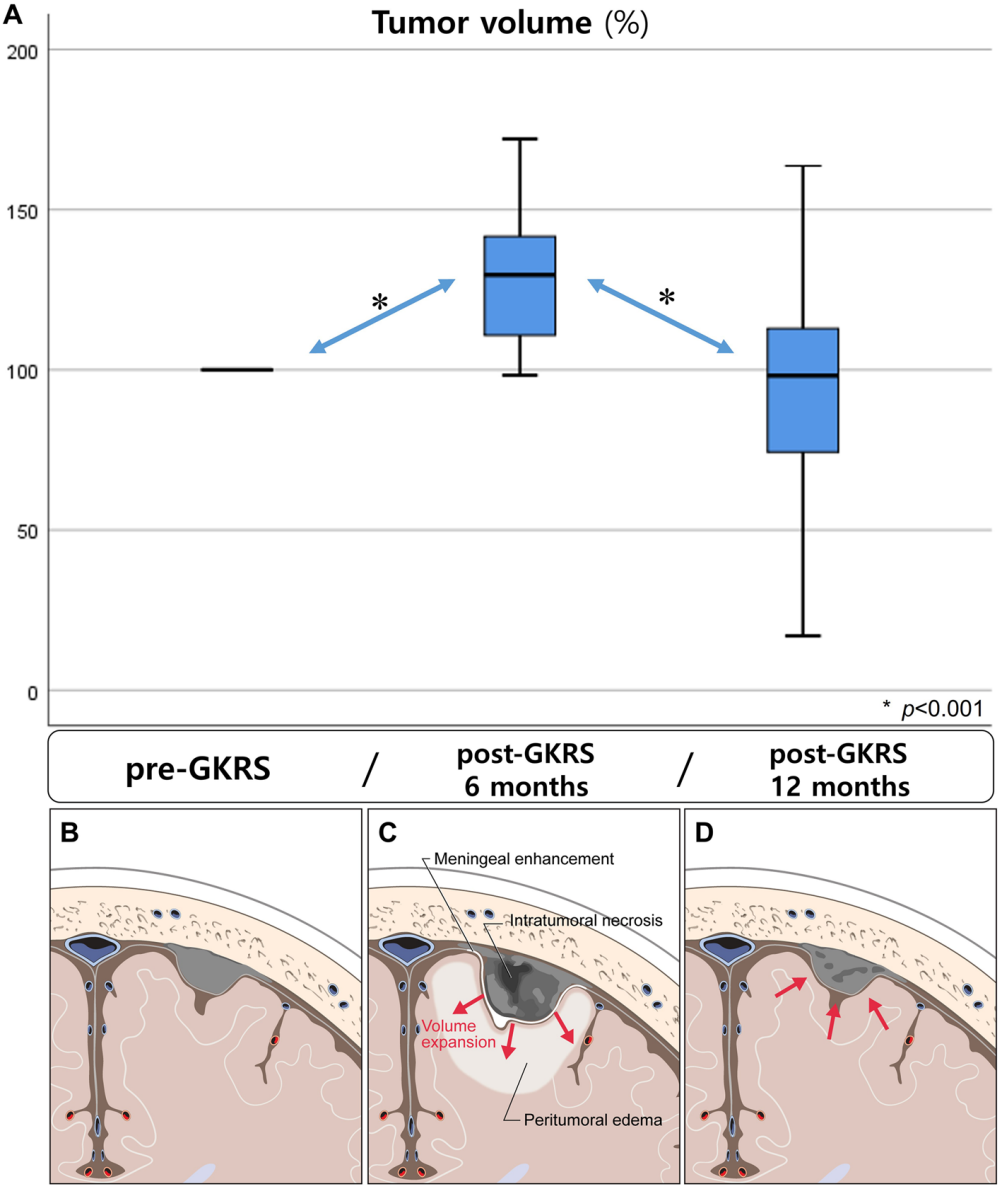
At 6 months after GKRS, the meningiomas with ITN have increased in volume by an average of 128.5%. And the tumors have a risk of PTE and meningeal enhancement (**C**). There is a statistically significant difference between the preoperative tumor volume and the tumor volume at 6 months (**A**, *p* < 0.001). However, by 12 months after the GKRS, the ITN has disappeared, and the volume is decreased to 94.6%. The PTE and meningeal enhancement also have disappeared (**D**). There is a statistically significant difference between the tumor volumes at 6 and 12 months (**A**, *p* < 0.001). *GKRS* gamma knife radiosurgery, *ITN* intratumoral necrosis, *PTE* peritumoral edema.

As shown in Table 3, the tumor volume was increased at the first follow-up in cases with ITN but not in cases without ITN. Based on the preoperative tumor volumes of the 34 meningiomas with ITN, the mean postoperative tumor volumes at 6 and 12 months were 128.5% and 94.6%, respectively. There were statistically significant differences between the preoperative tumor volume and the tumor volume at 6 months (*p* < 0.001) and between the tumor volumes at 6 and 12 months (*p* < 0.001) (Fig. 2). Of the 34 patients with ITN, 9 had PTE; 6 patients with PTE were asymptomatic, and only 3 patients with PTE showed neurological symptoms. In all patients, the symptoms and PTE improved at 6 months after conservative treatment.

## Discussion

This study on the occurrence of ITN and PTE following GKRS treatment for meningioma had two important findings. First, the study elucidated the natural course of meningioma with ITN after GKRS and presented evidence of pseudoprogression. Although pseudoprogression after radiosurgery in vestibular schwannoma has been reported previously^19–22^, pseudoprogression after radiosurgery in meningioma is a novel finding. By 6 months after GKRS, the meningiomas with ITN had increased in volume by an average of 128.5% and were associated with meningeal enhancement and a risk of PTE. However, by 12 months after the GKRS, the ITN had disappeared, the volume had decreased to 94.6%, and the PTE and meningeal enhancement had improved (Fig. 2). Therefore, a volume expansion accompanied by ITN within 12 months after GKRS for meningioma is likely to be pseudoprogression, which can be treated conservatively rather than by immediate surgical resection.

Second, our study found that the presence of ITN at 6 months after GKRS for meningioma was a risk factor for PTE, and that ITN was associated with a 70% dose volume. Previously, some articles have identified ITN as a risk factor for PTE^9,23^. Chen et al. presented two cases of meningioma after GKRS that were complicated with ITN and PTE^9^. Lee et al. argued that the maximum dose and target volume were significantly related to ITN, and that ITN and the maximum dose were significantly related to the development or aggravation of PTE^23^. In our study, we could not evaluate the effect of maximal dose on PTE, because most of the patients (98.4%) had the same prescription dose (14 Gy 50% marginal dose). However, our finding that ITN is significantly correlated to PTE is consistent with and confirms previous reports. Therefore, our study is the first large-scale study to verify that ITN is a significant risk factor for PTE.

**Figure 3:**
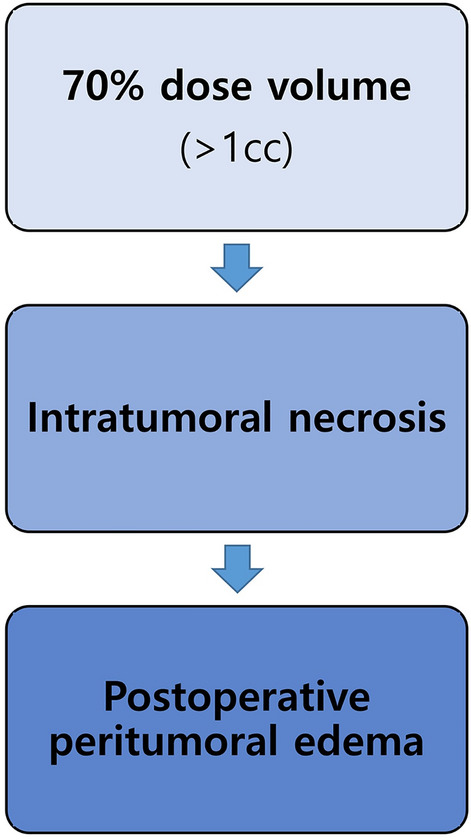
Flow chart of the progression from a 70% dose volume to ITN to PTE. We identified a 70% dose volume > 1 cc as a risk factor for ITN, and ITN as a risk factor for PTE. *ITN* intratumoral necrosis, *PTE* peritumoral edema.

In addition, we identified that a 70% dose volume > 1 cc is a risk factor for ITN, which can subsequently lead to PTE (Fig. 3). Although vasogenic edema is a major component of PTE in meningioma, cytotoxic edema is known to contribute to PTE as well^24–26^. It is known that the presence of PTE is strongly correlated with the expression of vascular endothelial growth factor (VEGF) within a meningioma^26–29^. Some argue that there is also a significant correlation between matrix metalloproteinase 9 (MMP-9) and the presence of PTE^30,31^. Such a protein-based pathophysiology for PTE would necessitate a partial breakdown of the tumor–brain interface^26,32^. Based on our findings, we suggest that ITN triggered by GKRS may play a role in the breakdown.

After GKRS for meningioma, PTE is a relatively common radiation-induced complication, and there have been many reports of PTE following GKRS. The incidence of symptomatic PTE after GKRS for meningioma has been reported as 5–11%^33–37^, whereas all PTE is 13–28%^23,34,36–39^. Our study showed a lower incidence of PTE than previously reported (2.4% for symptomatic PTE and 7.9% for all PTE). This low incidence of PTE is probably due to the nature of the tumors included in our study. It is already widely known that tumor volume and size are significant factors for PTE after GKRS for meningioma^8,33–37,40–42^. Although the tumor volume did not show statistical significance in our study, we do not deny that tumor volume is a significant factor for PTE after GKRS. Since the meningiomas included in our study were mostly small tumors (88.2% were < 3 cc), it is possible that the influence of tumor volume was underestimated.

While some studies claim that tumor location is a risk factor for PTE^41,43^, others argue the opposite^8,40,44^. Meningiomas differ in growth patterns and tumor shape depending on location. The contact area between the tumor and the brain parenchyma varies depending on the tumor shape. Studies on meningioma show that the ratio of the dural base area to the brain parenchymal contact area affects the radiosurgical outcome^8,14,37,42,45–47^. Therefore, in this study, we limited the study subject to convexity meningioma in order to remove the tumor location as a confounding variable and focus on the radiosurgical prescription parameters that we could control.

In our study, only 3 of 9 patients who had PTE with ITN showed neurological symptoms, and the symptoms disappeared after 6 months in all patients. Therefore, neurosurgeons should reassure their patients by providing sufficient explanations to patients with a high risk for PTE with ITN.

We faced certain limitations during the course of this study. This study was limited by its retrospective study design. Despite being the largest study regarding the occurrence of ITN after GKRS for meningioma, our study was conducted using a small sample size. In our study, only follow-up MRIs at 6 and 12 months after GKRS were included in the study. Further studies might elucidate the longer-term natural course of meningioma with ITN through long-term follow-up.

## Conclusions

When performing GKRS for meningioma, a 70% dose volume ≥ 1 cc is a risk factor for ITN. At 6 months after GKRS, meningioma with ITN is accompanied by a transient volume increase and PTE, which are characteristics of pseudoprogression. These characteristics improve at 12 months after GKRS. This study should help neurosurgeons plan and treat meningiomas using GKRS.

## Data Availability

The datasets used and analyzed during the current study available from the corresponding author on reasonable request.
